# A Janus-Faced Bacterium: Host-Beneficial and -Detrimental Roles of *Cutibacterium acnes*

**DOI:** 10.3389/fmicb.2021.673845

**Published:** 2021-05-31

**Authors:** Holger Brüggemann, Llanos Salar-Vidal, Harald P. M. Gollnick, Rolf Lood

**Affiliations:** ^1^Department of Biomedicine, Aarhus University, Aarhus, Denmark; ^2^Department of Clinical Microbiology, Fundacion Jimenez Diaz University Hospital, Madrid, Spain; ^3^Department of Dermatology and Venerology, Otto-von-Guericke University Magdeburg, Magdeburg, Germany; ^4^Division of Infection Medicine, Department of Clinical Sciences, Lund University, Lund, Sweden

**Keywords:** *Cutibacterium acnes*, acne (acne vulgaris), implant-associated infection, skin microbiome, beneficial bacteria, *Propionibacterium acnes*

## Abstract

The bacterial species *Cutibacterium acnes* (formerly known as *Propionibacterium acnes*) is tightly associated with humans. It is the dominant bacterium in sebaceous regions of the human skin, where it preferentially colonizes the pilosebaceous unit. Multiple strains of *C. acnes* that belong to phylogenetically distinct types can co-exist. In this review we summarize and discuss the current knowledge of *C. acnes* regarding bacterial properties and traits that allow host colonization and play major roles in host-bacterium interactions and also regarding the host responses that *C. acnes* can trigger. These responses can have beneficial or detrimental consequences for the host. In the first part of the review, we highlight and critically review disease associations of *C. acnes*, in particular acne vulgaris, implant-associated infections and native infections. Here, we also analyse the current evidence for a direct or indirect role of a *C. acnes*-related dysbiosis in disease development or progression, i.e., reduced *C. acnes* strain diversity and/or the predominance of a certain phylotype. In the second part of the review, we highlight historical and recent findings demonstrating beneficial aspects of colonization by *C. acnes* such as colonization resistance, immune system interactions, and oxidant protection, and discuss the molecular mechanisms behind these effects. This new insight led to efforts in skin microbiota manipulation, such as the use of *C. acnes* strains as probiotic options to treat skin disorders.

## Introduction

*Cutibacterium acnes* (*C. acnes*) is a Gram-positive member of the skin microbiota and as such, a very prevalent microorganism associated with humans. It is a lipophilic microorganism, and the most dominant bacterium in sebaceous, lipid-rich areas of normal human skin; in addition, it is also very abundant on moist and dry skin areas (Byrd et al., 2018). It is also found in several other organs and tissue sites, such as oral cavity, stomach, lung, urinary tract, and prostate (Sasaki et al., 1980; Shannon et al., 2006; Delgado et al., 2011; Davidsson et al., 2016); however, it is unclear if the organism can live and thrive in sites other than the skin or if its detection is a result from skin-derived carry-over, sample contamination, or a temporary breach.

**Figure 1:**
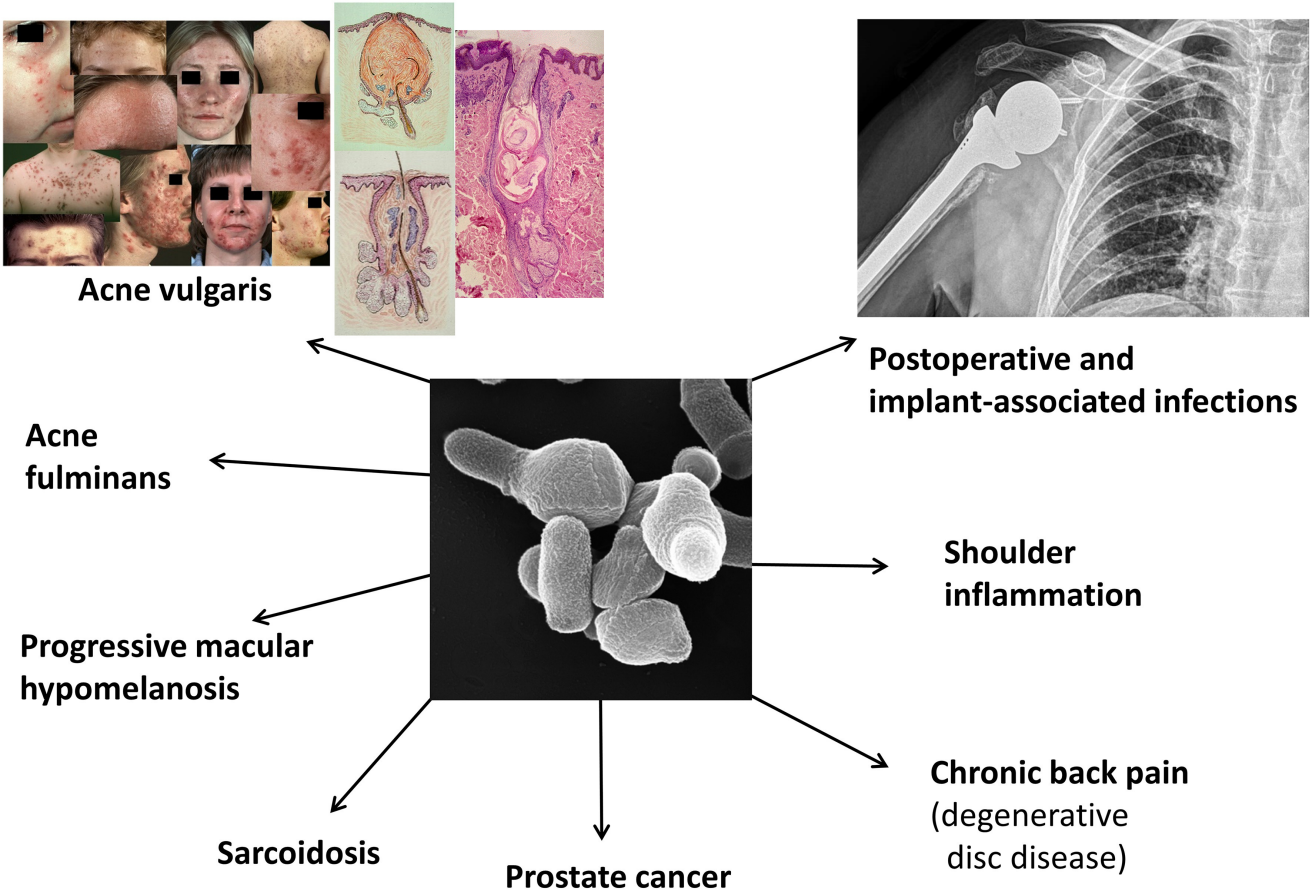
Known or suspected disease associations of *C. acnes*. Shown are currently investigated and debated disease associations of *C. acnes*. In this review we mainly focus on acne vulgaris, implant-associated infections, and a few native infections. The clinical pictures regarding acne vulgaris are taken from Gollnick and Zouboulis (2014).

The colonization of *C. acnes*, or rather the fact that human defense systems allow *C. acnes* to colonize the largest organ, suggests that the bacterium does not harm the human host, at least not under normal circumstances. *C. acnes* grows particular well in sebaceous rich areas; during the development of puberty, with increased sebaceous gland activity, *C. acnes* predominantly colonizes those skin areas with preference of infrainfundibula of sebaceous follicles (Leyden et al., 1998). The successful colonization by *C. acnes* could indicate that the bacterium actually has host-beneficial roles. In this review, we highlight some of the known and suggested beneficial functions of *C. acnes*. On the other hand, most, if not all, bacteria can cause harm to the human host under certain conditions such as in a predisposed or immunocompromised state. This also holds true for *C. acnes*, in particular if bacteria breach the skin surface and reach deeper tissue sites. However, we still know very little about the active roles that *C. acnes* might have in disease formation or progression; even for the skin condition acne vulgaris (AV) there are still open questions regarding the exact involvement of *C. acnes* on the molecular level. This review aims at a description and evaluation of some disease associations of *C. acnes* (Figure 1, Table 1).

**Table 1:** Disease associations of *C. acnes* and evaluation of the existing evidence.

**Disease**	Level of evidence^*^	**Phylotype association (enrichment)**	**Selected reviews**	**Selected primary literature**
Acne vulgaris	A Disease association Detection (CD, CI, IHC/IF)^**^ Skin model Animal model (with limitations) Responsive to antibiotic treatment	IA_1_ (SLST types A andC; possibly also SLST types D and E) and, possibly, IC (SLST type G)	Dréno et al., 2018, 2020; Platsidaki and Dessinioti, 2018; Brüggemann, 2019; McLaughlin et al., 2019	Lomholt and Kilian, 2010; McDowell et al., 2011; Fitz-Gibbon et al., 2013; Dagnelie et al., 2018
Progressive macular hypomelanosis	B Disease association Detection (CD and CI)^**^ Responsive to antibiotic treatment	III (SLST type L)	McDowell et al., 2020	Barnard et al., 2016; Petersen et al., 2017
Implant associated infections PJI Cardiac devices Neurosurgical shunts Breast implants	A Disease association Detection (CD and CI)^**^ Animal model Responsive to antibiotic treatment	Conflicting results: II (SLST type K) IB (SLST type H) IA_1_ (SLST type A)	Portillo et al., 2013; Achermann et al., 2014; Aubin et al., 2014; Gharamti and Kanafani, 2017; Boisrenoult, 2018; Renz et al., 2018	Zeller et al., 2007; Aubin et al., 2017; Banzon et al., 2017; Lavergne et al., 2017; Liew-Littorin et al., 2019; Lee et al., 2020; Suzuki et al., 2020a
Spine instrumentation infections Spine osteomyelitis	B Disease association Detection (CD)^**^ Responsive to antibiotic treatment (with limitations)	IA_1_ (SLST type A) IA_2_ (SLST type F)	Khalil et al., 2019	Uçkay et al., 2010
Disc degeneration and Modic type 1 changes	B Disease association Detection (CD, CI, IHC/IF)^**^ Animal model Responsive to antibiotic treatment (with limitations)	IA_1_ (SLST type A) II (SLST type K)	Capoor et al., 2019; Manniche and O'Neill, 2019; Jha and Sairyo, 2020	Albert et al., 2013; Capoor et al., 2017; Lin et al., 2018; Ohrt-Nissen et al., 2018
Sarcoidosis	B Disease association Detection (CD, CI, IHC/IF)^**^ Animal model	?	Eishi, 2013; Yamaguchi et al., 2021	Nagata et al., 2017; Werner et al., 2017; Suzuki et al., 2018; Song et al., 2019; Beijer et al., 2021
Prostate cancer	C Disease association Detection (CD, IHC/IF)^**^ Animal model	II (SLST type K)	Brüggemann and Al-Zeer, 2020	Cohen et al., 2005; Alexeyev et al., 2007; Fassi Fehri et al., 2011; Shinohara et al., 2013; Bae et al., 2014; Davidsson et al., 2016, 2017

* *A, B, C: strong, medium, and weak evidence, respectively, based on the existing scientific literature*.

** *CD, culture-dependent; CI, culture-independent; IHC/IF, immunohistochemistry/immunofluorescence*.

In the last decades, improvement of technologies such as next generation sequencing (NGS) has provided new possibilities to study *C. acnes* and the skin microbiome. Full genome sequencing of many different strains has highlighted the pan-genome and the genetic repertoire of *C. acnes* (Tomida et al., 2013; Scholz et al., 2016). This has led to the identification of host-interacting, secreted, and/or surface-exposed proteins of *C. acnes* as well as other molecules and metabolites. This review contains current knowledge of bacterial factors thought to be important in host-beneficial or -detrimental functions. In addition, sequencing of almost 300 genomes (status February 2021) has provided a deep insight into the population structure of this species. Results of the phylogenomic analysis and the comparison with genomes of other propionibacteria, in particular the classical diary propionibacteria, e.g., *Propionibacterium freudenreichii*, have led to a proposal of changing the species name from *Propionibacterium acnes* to *Cutibacterium acnes* (Scholz and Kilian, 2016). This name change provoked a controversy among scientists; among others, it was noted that a new species name might generate some confusion among clinicians (Alexeyev et al., 2018).

**Figure 2:**
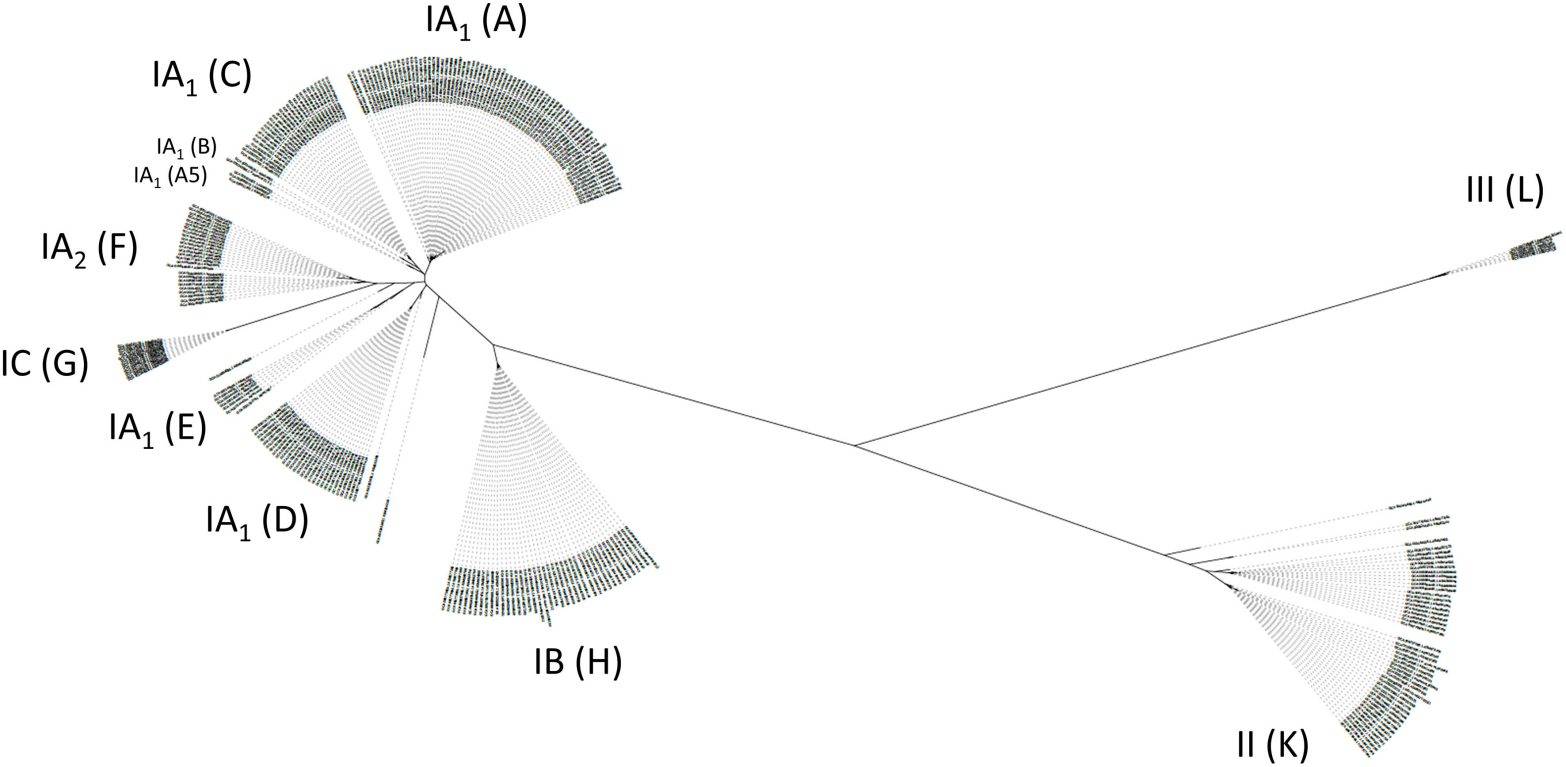
Diversity of the population of *C. acnes*. A population tree is shown based on a phylogenomic comparison relying on core genome-located nucleotide polymorphisms. Distinct phylotypes of *C. acnes* are highlighted as six main phylotypes IA_1_, IA_2_, IB, IC, II, III, and 10 SLST types A-L. SLST types F, G, H, K, L correspond to phylotypes IA_2_, IC, IB, II, and III, respectively. Please note that SLST types A–E are distinct clades of phylotype IA_1_. In particular, SLST types D and E are distinct from acne-associated SLST types A–C. In total, 286 publicly available *C. acnes* genomes were analyzed and the closed genome of strain KPA171202 (phylotype IB, SLST type H) was taken as reference [modified from Brüggemann (2019)].

Among *C. acnes*, we distinguish six main phylotypes, often labeled as IA_1_, IA_2_, IB, IC, II, and III (McLaughlin et al., 2019). Several typing schemes to distinguish strains of *C. acnes* exist, traditionally based on multi-locus sequence typing (MLST) schemes and now more and more replaced by whole genome-based phylotyping (Lomholt and Kilian, 2010; McDowell et al., 2011; Scholz et al., 2014). Three subspecies are currently distinguished, subspecies *acnes* (comprising phylotypes IA_1_, IA_2_, IB, IC), subspecies *defendens* (phylotype II) and subspecies *elongatum* (phylotype III) (Dekio et al., 2019). Based on the core genome phylogeny of *C. acnes*, a single-locus sequence typing (SLST) scheme was created that can differentiate 10 lineages in total (types A-L); the complex phylotype IA_1_ is further split into five SLST types (A-E) (Scholz et al., 2014). The other SLST types correspond to the phylotypes as follows: F, IA_2_; G, IC; H, IB; K, II; L, III (Figure 2). This review will often refer to the main six phylotypes (and the 10 SLST types) and their distinct associations with health and disease. Apart from core genome differences, the accessory genome of *C. acnes* is relatively small but comprises elements such as a linear plasmid (designated p15.1.R1 or pIMPLE-HL096PA1, encoding a conjugation apparatus and a tight adherence pili locus) and a circular plasmid (designated pTZC1, encoding genes conferring resistance to macrolides, clindamycin and tetracycline) (Tomida et al., 2013; Davidsson et al., 2017; Aoki et al., 2020). In addition, around 60 other regions that are not part of the core genome can be identified in the pan-genome of *C. acnes*; these non-core genes are often, but not exclusively, phylotype-specific, and code for a variety of functions such as different transport systems, bacteriocin synthesis, resistance to heavy metals and antibiotics, restriction modification systems, CRISPR/cas systems, (cryptic) prophages, transposases, carbohydrate and amino acid processing enzymes, and many so far unknown functions (Brüggemann et al., 2012; Tomida et al., 2013; Scholz et al., 2016).

## Mr. Hyde—The Pathogenic Side Of *C. acnes*: Proven And Suggested Disease Associations And Mechanisms

### General Considerations

Hundreds of studies have been carried out to shed light on the potential role of *C. acnes* in various diseases. Due to the ubiquitous bacterium's presence on human skin (Byrd et al., 2018), many efforts focused on skin disorders, in particular AV. Mainly in the last two decades several non-skin diseases were reported to be associated with *C. acnes*, including implant-associated infections (IAIs), primary joint and bone infections, sarcoidosis, and prostate cancer (Figure 1).

Here, we do not aim at the presentation of the entire literature that reported evidence and counter-evidence for a role of *C. acnes* in disease formation and/or progression; rather, this review will present and discuss selected disease associations of *C. acnes*. The selection is based on the availability of recent advances, as well as new findings that shed light on *C. acnes*' role and its host-interacting properties.

### Skin Disorders Associated With *C. acnes*

#### *C. acnes* and Acne Vulgaris

Historically, much effort has been undertaken to pinpoint the exact role of *C. acnes* in the onset and/or progression of AV. Many hypotheses and possible mechanisms have been suggested, but solid evidence is still scarce. On the other hand, there is no case of AV without *C. acnes*. There is strong evidence that therapeutically induced reduction of *C. acnes'* population (e.g., directly by antibiotics or indirectly by retinoids that suppress sebum production) diminish inflammation. The reader is referred to a few recent reviews that summarize diverse aspects on the debated role of *C. acnes* in AV (Dréno et al., 2018, 2020; Platsidaki and Dessinioti, 2018; Brüggemann, 2019; McLaughlin et al., 2019).

Here, a few selected features regarding *C. acnes*' association with AV should be highlighted. In the last decade, the concept of a dysbiosis of the *C. acnes* population on acne-affected skin compared to normal skin was put forward; such a shift of the *C. acnes* population was -to some extent- observed with culture-dependent and culture-independent methods (Lomholt and Kilian, 2010; McDowell et al., 2011; Fitz-Gibbon et al., 2013; Dréno et al., 2020). In these studies, it was stated that strains belonging to certain phylotypes of *C. acnes* are AV-associated, while other strains belonging to different phylotypes are preferentially associated with healthy skin. More accurately, in case of AV-affected skin compared to normal skin, a higher relative abundance of certain strains, in particular strains of phylotype IA_1_ (and phylotype IA_2_) was observed. However, strains of phylotype IA_1_ are, in average, also more abundant on healthy (facial) skin than strains belonging to the other phylogenetic lineages, i.e., phylotypes IA_2_, IB, IC, II, and III (McLaughlin et al., 2019). So far, not many studies have used the SLST typing scheme to determine the phylogeny of acne-associated strains. A recent study has reported the association of the SLST type A (a subset of IA_1_ strains, Figure 2) with acne-affected skin; however, this study only processed a rather small cohort (*n* = 36) (Dagnelie et al., 2018). In contrast, studies with Japanese patients have detected the SLST type F (IA_2_) as strongly acne-associated (Nakase et al., 2017, 2020), suggesting geographic differences between Europe and Asia. In addition, the SLST type C (IA_1_) is enriched among acne patients; this SLST type corresponds to the (MLST-based) phylogenetic lineage CC3 (Lomholt and Kilian, 2010; McLaughlin et al., 2019).

It is fairly unlikely that phylotype IA_1_ strains (or rather strains of SLST types A and C) or phylotype IA_2_ strains (SLST type F) isolated from acne-associated skin have *per se* different properties than phylotype IA_1_/IA_2_ strains isolated from healthy skin. In line with this, a study found no differences in the (core as well as accessory) genomes of phylotype IA_1_ strains isolated from acne and healthy skin, respectively (Lomholt et al., 2017). Moreover, there is no solid evidence that supports the assumption that phylotype IA_1_/IA_2_ strains are *per se* more virulent compared to strains of other phylotypes, even though such differences on the strain level might exist. Studies that have compared strain properties of *C. acnes* (comparing either strains belonging to the same phylotype or strains belonging to different phylotypes) regarding their pathogenic potential came to conflicting results (Nagy et al., 2005; Jasson et al., 2013; Lheure et al., 2016; Agak et al., 2018). Current studies that report strain comparisons need to be handled with caution; much can depend on the individual, selected strains (that can have slight differences, e.g., in growth behavior and oxygen sensitivity) as well as on the method/model that is applied to evaluate bacterial pathogenicity or host-interaction.

**Figure 3:**
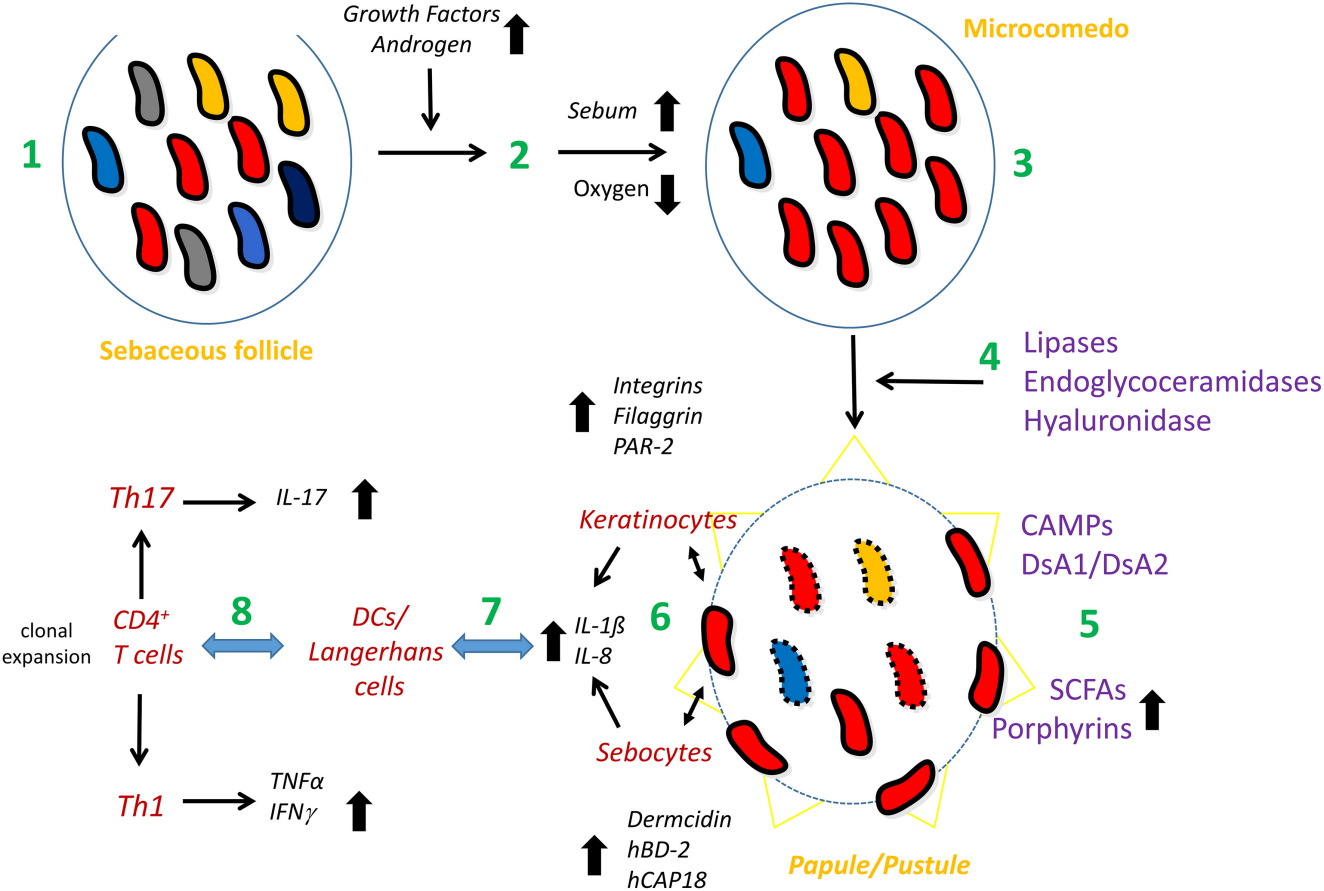
Model of the possible involvement of *C. acnes* in acne vulgaris. The healthy pilosebaceous unit is colonized with a mixture of different *C. acnes* phylotypes/strains (1). Androgen and growth hormone levels in the puberty age rise; these activate sebaceous glands to produce more sebum (2). Exceeding sebum and hyperkeratinization lead to the clogging of the sebaceous duct, the infrainfundibulum, and therefore, to microcomedo formation (acne precursor lesion). The follicular homeostasis is disrupted; the microenvironment of the microcomedo is more anaerobic, providing an advantage and/or disadvantage for different *C. acnes* phylotypes/strains, resulting in the predominance of type IA_1_ (SLST types A and C) and/or IA_2_ (SLST type F) strains in the comedo (3). Type IA_1_/IA_2_ strains produce and secrete host-tissue degrading enzymes such as a type IA-specific hyaluronidase, endoglycoceramidases and lipases; the latter lead to the accumulation of free fatty acids (4). In addition, type IA_1_/IA_2_ strains produce CAMP factors (CAMP1 and 2) as secreted and cell surface-attached proteins and the adhesive surface glycoproteins dermatan-sulfate adhesins/fibrinogen-binding proteins (DsA1/DsA2). They also secrete short-chain fatty acids such as propionate and produce porphyrins at higher levels (5). These bacterial properties further modulate the follicular microenvironment and pave the way for a closer contact of the bacterium with the cellular microenvironment of the follicle (6), including keratinocytes and, possibly, sebocytes. The direct (bacterial surface) or indirect (secreted factors) contact of *C. acnes* with these cells activates a local innate immune response, mainly in a TLR2-dependent manner, resulting in the release of chemokines/cytokines, such as IL-8, IL-1β and others, and can also lead to the production of defense factors (6). Skin-resident DCs/Langerhans cells and macrophages sense and/or are recruited to and infiltrate the irritated tissue site (7). They also interact with skin-resident CD4^+^ T cells, resulting in clonal expansion (*C. acnes* clone-specific proliferation of T-cells). Mixed Th1/Th17 responses result in the secretion of other cytokines including IFN-γ and IL-17 (8). Together, this leads to the formation of papules and pustules as seen in inflammatory acne.

The relative enrichment of phylotype IA_1_/IA_2_ strains in acne could also have another explanation: the reduction of strains belonging to other phylotypes. An overall reduction of *C. acnes* strain diversity could be associated with AV; this implies that not one or a few *C. acnes* phylotypes are disease-associated but rather the lack of strain/phylotype diversity. However, a clear-cut, solid large scale study with 100's of early-stage acne patients and matched healthy controls is lacking; such a study should also take possible confounders into consideration, such as past and current treatments, since most acne suffers use some sort of (topical) treatment. A recent study indicated that loss of strain/phylotype diversity might go along with increased innate immune stimulation: a three-strain mixture (strains of phylotypes IA_1_, II, and III) elicited a weaker innate immune response in healthy skin explants than the three strains applied individually (Dagnelie et al., 2019). Regarding bacterial properties and factors that could influence the formation and/or progression of AV, some of them will be presented below, including CAMP factors, biofilm formation, porphyrin, and short-chain fatty acid production. Figure 3 summarizes a tentative model of the role of *C. acnes* in AV.

Taken together, the concept of an acne-associated *C. acnes* dysbiosis on the phylotype/strain level leading to a diversity loss seems likely but needs further proof. If the reduction of phylotype/strain diversity in acne-affected skin holds true, as current data suggests, it would imply that a diverse *C. acnes* population is a marker of healthy skin and such diversity thus may be beneficial for skin heath. The question then remains how a high strain diversity could contribute to health, e.g., which mechanisms are at play. Since different phylotypes of *C. acnes* are reported to have different microbiological, and -to some extent- different host-interacting properties (Nagy et al., 2005; Jasson et al., 2013; Lheure et al., 2016; Agak et al., 2018; Dekio et al., 2019), it can be speculated that a diverse *C. acnes* population can compensate or dampen phylotype-specific peculiarities, metabolize different components, provide complementary beneficial traits and/or undergo some sort of symbiotic relationship.

#### Other Skin-Disease Associated With *C. acnes*

*C. acnes* is suspected to be involved in other skin diseases. The disease progressive macular hypolmelanosis (PMH) is characterized by non-scaly hypopigmented skin areas that are visible in the sebaceous areas; often the lower back skin is affected. Regarding the role of *C. acnes* in PMH the reader is referred to a recent review (McDowell et al., 2020) that, among others, summarized and discussed data from primary studies (Barnard et al., 2016; Petersen et al., 2017). In brief, a rather clear enrichment of certain strains of *C. acnes* was seen in PMH lesions; these strains belong to phylotype III (SLST type L) of *C. acnes*. This phylotype (renamed to *C. acnes* subsp. *elongatum*) is poorly investigated so far; it is quite different from the other main phylotypes, also regarding its morphology (Dekio et al., 2019).

*C. acnes* might also play a role in acne fulminans, a rare, severe form of inflammatory acne, associated with painful ulceration, and in some cases with systemic signs. A recent study identified the SLST type A (phylotype IA_1_) in about 60% of patients (Bocquet-Trémoureux et al., 2020); however, as discussed above, the SLST type A is often found to be the dominating type in healthy skin as well. Thus, at present, there is no strong evidence that (a specific phylotype of) *C. acnes* is a driver of disease.

### Non-skin Diseases Associated With *C. acnes*

#### *C. acnes* and Implant-Associated Infections

More and more studies report the detection of *C. acnes* in IAIs. A few reviews have summarized the current knowledge regarding the role of *C. acnes* in IAIs (Portillo et al., 2013; Achermann et al., 2014; Aubin et al., 2014; Gharamti and Kanafani, 2017; Boisrenoult, 2018; Renz et al., 2018; Lin et al., 2020). It seems that *C. acnes* is now more often found in IAI-associated specimens than two decades ago. The reason for this increase is most likely related to changed procedures and diagnostic tools. One important change is the introduction of sonication of removed periprosthetic tissue specimens or removed medical devices before microbial cultivation (Trampuz et al., 2007). This technique is nowadays more often used in everyday clinical microbiology practice in many hospitals; it has been shown to increase the bacterial recovery from specimens (Trampuz et al., 2007; Esteban et al., 2008). In addition, cultivation procedures have been adapted to guarantee also the reliable detection of slow-growing anaerobic bacteria such as *C. acnes*; e.g., cultivation times have often been extended to 14 and even 21 days (Bossard et al., 2016; Kvich et al., 2016). Moreover, due to the introduction of MALDI-TOF mass spectrometry for bacterial species determination in the last decade, the identification of cultivated bacteria has become more easily, and samples with polymicrobial growth can be analyzed more comprehensively. Furthermore, the use of multiplex PCR techniques has also aided to improve the detection of low-virulent microorganisms such as *C. acnes* (Morgenstern et al., 2018; Sigmund et al., 2019).

##### Prosthetic Joint Infections

*C. acnes* is associated with around 10% of all prosthetic joint infections (PJIs), being more frequently isolated in late-chronic infections (Benito et al., 2019; Triffault-Fillit et al., 2019). The shoulder is the most frequent site of isolation, probably due to the greater rate of colonization in the axillary region than in the hip or knee (Boisrenoult, 2018; Lin et al., 2020). It is now estimated that *C. acnes* accounts for 31–70% of all PJIs after shoulder arthroplasty (Fink and Sevelda, 2017). *C. acnes* is also believed to be one of the main opportunistic pathogens involved in latent postoperative infections in spinal instrumentation surgeries (Khalil et al., 2019).

The diagnosis of *C. acnes*-associated PJI is difficult, mainly due to the lack of specific local inflammatory signs at the surgical site of infection. Persistent unexplained pain is often the only symptom; evidence of implant loosening is seen in a great number of cases (Shah et al., 2015; Lin et al., 2020). Even though *C. acnes* is primarily associated with low-grade, chronic or delayed infections, it can also cause acute infections (Zeller et al., 2007; Dodson et al., 2010). Systemic symptoms, such as fever are not frequent and inflammatory markers are normal or slightly elevated. Only half of the patients presented local signs of infections (Zeller et al., 2007; Lavergne et al., 2017). This might explain why *C. acnes* can be unexpectedly found in revision surgery (Benito et al., 2019). Clinical signs of infection are more often encountered during the first two years after arthroplasty procedure (Zeller et al., 2007). Nodzo et al. reported that shoulder PJIs present with a less robust host peripheral inflammatory response in comparison with knee and hip PJIs (Nodzo et al., 2017). Rates of infections are higher in males (Figa et al., 2017; Renz et al., 2018). One reason might be related to gender-specific differences regarding the distribution and frequency of hair follicles (Hudek et al., 2016). Abnormal radiographic findings are usually observed in a minority of cases of shoulder PJIs (Piggott et al., 2015; Shields et al., 2016). It is still unclear if humeral loosening may indicate a *C. acnes* infection. Radiographic findings (e.g., in *C. acnes*-associated spinal infections) may show the formation of a “halo” around screws, osteolysis or evidence of pseudoarthrosis of the fusion mass (Khalil et al., 2019).

##### Cardiac Device-Related Infections

*C. acnes* has also been described as a causative agent of cardiovascular device-related infections, involving prosthetic heart valves, permanent pacemakers, prosthetic valve rings, or implantable cardioverter-defibrillators. Diagnosis of *C. acnes*-associated infective endocarditis (IE) is complicated, due to the indolent nature of the infection, the slow growth rate of the microorganism and its consideration as a common contaminant in blood cultures (Clayton et al., 2006). In the *C. acnes*-associated IE cohort of Banzon et al. invasive disease was reported in 78% of the cases and embolic complications in 36% (Banzon et al., 2017), whereas in the study of Sohail et al. myocardial abscesses occurred in 36% of the cases (Sohail et al., 2009). Extended incubation of blood cultures is recommended to increase the microbiological yield. Banzon et al. showed that valve sequencing may aid in the identification of *C. acnes*-associated IE, in particular in culture negative cases (Banzon et al., 2017).

##### Breast Implant Infections

Capsular contracture is a frequent and severe complication following breast implantation. The etiology is not fully understood, but studies that employed sonication of the implant, have suggested that bacterial colonization is associated with capsular contracture (Reischies et al., 2017). Tamboto et al. demonstrated in a porcine model a relation between subclinical infection, bacterial biofilm formation and capsular contracture (Tamboto et al., 2010). The most common bacteria isolated in these samples are microorganisms from the skin microbiota, being *C. acnes* one of the most recovered ones (del Pozo et al., 2009; Karau et al., 2013). In the study of Lee et al. *C. acnes* was found as the most prevalent microorganism in cases with chronic infection (Lee et al., 2020).

##### Neurosurgical Shunt Infections

*C. acnes* is considered an emerging opportunistic pathogen in neurosurgery procedures (Nisbet et al., 2007). It may be responsible for ~15% of infections associated with shunt tubular devices, that drain cerebrospinal fluid (CSF) from cerebral ventricles to other body sites, usually the peritoneum (Conen et al., 2008; Bayston et al., 2010). Clinical symptoms in shunt infections are non-specific; the absence of fever is common (Aubin et al., 2014). PCR has been described as effective method to detect *C. acnes* in CSF infections (Suzuki et al., 2020a), although this method might be too sensitive.

##### Molecular Typing of *C. acnes* Isolates From IAIs

Sequence types (ST) or clonal complexes (CC) have been determined for a large population of *C. acnes-*associated IAI strains in order to establish an association between certain phylotypes and infection. It has been shown that phylotypes IB and II were more frequently isolated from PJIs (McDowell et al., 2012; Aubin et al., 2017). Regarding the CCs, Aubin et al. described CC36 (phylotype IB, SLST type H) and CC53 (phylotype II, SLST type K) as predominant in their cohort. In contrast, in the study of Littorin et al. CC18 (phylotype IA_1_, more specifically SLST type A) was found to be predominant, followed by CC53 (Liew-Littorin et al., 2019). The study of El Sayed et al. found that CC18 was the most abundant CC, followed by CC36 (el Sayed et al., 2019). In spine instrumentation infections, *C. acnes* strains mostly belonged to phylotypes IA_1_ (CC18) and IA_2_ (CC28; SLST type F) (Aubin et al., 2017). Taken together, these results are contradictory and might indicate that there is no predominate PJI-specific phylotype, implying that all *C. acnes* types might have the potential to cause PJIs and it rather depends on the individual strain.

##### *C. acnes* in IAIs: True Etiology or Sample Contamination?

*C. acnes* is considered a low-virulent microorganism that causes infections with subtle clinical presentation, making positive cultures for *C. acnes* in IAIs difficult to interpret. Indeed, the significance of its detection is not always clear because it is considered a common contaminant due to its omnipresence in man-made environments. It has also been reported that *C. acnes* is a possible commensal of the human shoulder joint (Hudek et al., 2020), fueling further the debate whether it represents a contaminant, a passive/transient colonizer or bystander or whether it accounts for true infection. Given the high rates of positive cultures after primary and secondary shoulder arthroplasty, Namdari et al. demonstrated that the most frequently isolated microorganisms from shoulder revision arthroplasty (*C. acnes* and coagulase-negative staphylococci) are also the most common bacterial contaminants from air in the operating room (Namdari et al., 2020). A careful interpretation of the culture results is needed to distinguish a true-positive from a false-negative result. Parameters, like the number of culture-positive specimens per patient, the cultivation time, and the colony-forming unit (CFU) count help to make this distinction. Frangiamore et al. and Salar-Vidal et al. suggested that clinically relevant isolates of *C. acnes* need a shorter incubation period in comparison with probable contaminants (Frangiamore et al., 2015; Salar-Vidal et al., 2020). On the other hand, a long incubation time could be needed to reactivate dormant cells of *C. acnes* and/or intracellular or biofilm-embedded *C. acnes*. However, not much is currently known about such different states of *C. acnes*.

It is widely accepted that the number of culture-positive specimens per patient is a measure to evaluate the probability of infection (Bémer et al., 2008; Asseray et al., 2010; Frangiamore et al., 2015). In addition, the CFU count may be taken into consideration, as contaminants in most cases often result in very low CFU counts (Esteban et al., 2013; Burnham et al., 2017; Salar-Vidal et al., 2020). In fact, the use of implant sonication fluid alone could increase the rate of false positives if no CFU threshold is established (Grosso et al., 2018). However, neither long cultivation times nor low CFU counts serve as definitive markers of contamination. Each patient should be evaluated individually and interpretations should be done in combination with other relevant clinical and histopathological findings.

#### Native Infections by *C. acnes*

##### Spine Infections

*C. acnes* has been rarely associated with native infections of the spine. Native spine osteomyelitis or spondylodiscitis remains difficult to diagnose. Patients with an infection potentially caused by *C. acnes* usually present with lower back pain without any associated systemic symptoms, and levels of inflammatory markers, such as C-reactive protein, are normal or slightly elevated (Khalil et al., 2019). Magnetic resonance imaging is needed to confirm the diagnosis, and if possible, at least two positive tissue cultures should be obtained. The major risk factor is a past history of spine surgery with an average of 34 months between the procedure and the onset of symptoms (Uçkay et al., 2010). In most cases, these patients have a favorable outcome with antibiotic therapy alone (Kowalski et al., 2007).

Certain controversy exists regarding the role of *C. acnes* in the pathogenesis of intervertebral disc degeneration and Modic type 1 changes of disc atrophy (fissuring and edema of the endplates) in herniated discs. In 2001, the study of Stirling et al. was the first to report positive cultures of *C. acnes* in intervertebral disc material after microdiscectomy (Stirling et al., 2001). Since then, several studies have reported the identification of *C. acnes* in patients with degenerative disc disease (Albert et al., 2013; Capoor et al., 2017, Ohrt-Nissen et al., 2018; Jha and Sairyo, 2020), although other studies do not support the true presence of *C. acnes* inside discs; if found it is often regarded as contaminant (Chen et al., 2016). Capoor et al. demonstrated the presence of a *C. acnes* biofilm in resected intervertebral discs by fluorescent in-situ hybridization (Capoor et al., 2017). In addition, Lin et al. showed that *C. acnes* may be involved in intervertebral disc degeneration by inducing apoptosis of nucleus pulposus cells *via* the TLR2/JNK pathway (Lin et al., 2018). The identification of *C. acnes* may indicate a true etiology in the pathogenesis of Modic type I changes, which could be responsive to antibiotic treatment, even though it is not possible to prove retrospectively which of those patients were truly infected. Further research and clinical trials are needed to clarify the infectious nature of *C. acnes* in this disease.

##### Prostate Pathologies

A recent review summarized the current evidence level of the involvement of *C. acnes* in prostate inflammation and prostate cancer (Brüggemann and Al-Zeer, 2020). There is relatively little doubt that *C. acnes* can be cultivated from a considerable portion of biopsy specimens, mainly obtained from radical prostatectomy. However, there is little knowledge about the exact origin of the recovered bacteria, i.e., if they represent contamination/carry-over from the urogenital tract, or if they are colonizers of the (anoxic regions) of the tumor tissue. For instance, a study from Sweden reported that *C. acnes* was cultured in 60% of the prostate cancer cases (*n* = 100) and in 26% of cancer-free controls (*n* = 50) (Davidsson et al., 2016). The phylotype II (SLST type K) was the most dominant type among *C. acnes* strains obtained from prostatic tissue and 26% of those strains carried an extrachromosomal element (Davidsson et al., 2017). In contrast, a French study has detected only very few *C. acnes* positive samples in their cohort (*n* = 36) (Bidaud et al., 2020); the biopsy procedure involved antibiotic prophylaxis that could explain the relative little bacterial recovery. Recent studies aimed at the determination of the urogenital tract (tumor) microbiome using culture-independent NGS-based techniques (Shrestha et al., 2018; Banerjee et al., 2019). Unfortunately, such low biomass studies often exclude *C. acnes* due to its notorious presence in control samples (Mollerup et al., 2016; Walker et al., 2020). Thus, NGS-based microbiome studies are unlikely to contribute significantly to the open questions regarding the presence and possible role of *C. acnes* in the prostate microenvironment.

##### Sarcoidosis

Sarcoidosis is a systemic inflammatory disease, characterized by the formation of non-necrotizing granulomas; these are most often detected in the lungs, but can be found also in the skin, lymph nodes, eyes, and other body sites and organs. The cause of sarcoidosis is unknown; infectious and non-infectious agents, individually or in combination, could play a role. Bacterial candidates as infectious causes of sarcoidosis are primarily *Mycobacterium tuberculosis* and *C. acnes* (Eishi, 2013). A recent review summarizes current insights in the disease contribution of *M. tuberculosis* and *C. acnes* to sarcoidosis (Yamaguchi et al., 2021). Several recent studies have identified *C. acnes* in granulomas of sarcoidosis patients; detection was achieved with DNA-based techniques, but also with immunohistochemistry/immunofluorescence staining (Nagata et al., 2017; Suzuki et al., 2018; Beijer et al., 2021). So far, no specific *C. acnes* phylotype has been associated with sarcoidosis. The involvement of *C. acnes* in sarcoidosis is also supported by experiments in mice (Werner et al., 2017; Song et al., 2019). As a possible disease scenario, invasive *C. acnes*, possibly derived from the skin, could result in a latent, intracellular state, for example in macrophages. Intracellular presence and/or proliferation could activate immune responses, mediated by bacterial factors, followed by the formation of insoluble immune complexes, in particular in individuals predisposed with a Th1 hypersensitivity against *C. acnes* (Yamaguchi et al., 2021). The multifaceted interactions of *C. acnes* with the immune system and relevant machineries such as autophagy are presented in the chapter Sensing and Signalling of Extra- and Intracellular *C. acnes* and Innate and Adaptive Immune Responses.

### *C. acnes* Virulence Factors and Traits

A few bacterial products and traits that possibly could influence disease pathologies have been investigated. These include e.g., CAMP (Christie–Atkins–Munch-Petersen) factors, dermatan-sulfate adhesins (DsAs), lipases, sialidases, hyaluronidases, putative endoglycoceramidases, porphyrins, short-chain fatty acids (SCFAs), cell wall polysaccharide/lipoglycan, and lipoproteins (Brüggemann et al., 2004; Valanne et al., 2005; Lodes et al., 2006; Holland et al., 2010; Mak et al., 2013; Yu et al., 2016; Brüggemann, 2019; McLaughlin et al., 2019). Some of these factors are actively or passively secreted, others are part of the cell surface or attached to/embedded into the cell wall. A recent study has analyzed a sortase of *C. acnes*, an enzyme important for cell wall attachment of secreted proteins. The study predicted 19 sortase substrates, among them some of the above-mentioned factors, including CAMP factor 1 (locus tag: PPA1340), sialidases (PPA1560, PPA1821), and DsAs (PA2127, PPA2210) (Girolamo et al., 2019).

The evidence level of the involvement of the above-mentioned factors in disease pathology is incomplete. In fact, a clear-cut virulence factor of *C. acnes* has yet to be identified, since there is very little insight into their functionalities. The lack of functional data can be partially explained with the lack of an efficient gene knock-out system for *C. acnes*; so far only very few mutant strains of *C. acnes* were created (Sörensen et al., 2010; Allhorn et al., 2016; Nazipi et al., 2017). Another reason is the apparent redundancy of host-interacting factors; *C. acnes* possesses multiple copies of genes for CAMP factors, DsAs, lipases, sialidases, putative endoglycoceramidases, and lipoproteins (Brüggemann et al., 2004). The redundancies indicate their importance for colonization and survival of *C. acnes* on human skin. There are only limited sequence differences regarding the mentioned host-interacting factors between the different *C. acnes* phylotypes. As an exception, substantial sequence differences can be detected for the hyaluronidase: there are two variants present in *C. acnes*. One variant is present in phylotype IA strains and the other variant is found in strains of phylotypes IB and II (Nazipi et al., 2017).

Here, we will briefly introduce and discuss new data regarding bacterial factors and traits that are suspected to be important for bacterial pathogenesis, in particular in AV, namely the CAMP factors, lipases and the metabolites/molecules SCFAs and porphyrins. We will also briefly discuss the evidence for biofilm formation of *C. acnes*, and its relevance in IAIs.

#### CAMP Factors

A review has summarized knowledge regarding CAMP factors in *C. acnes* (McDowell et al., 2013). There are five CAMP factor homologs (CAMP1-CAMP5) present in (all phylotypes of) *C. acnes*. In particular, CAMP1 and CAMP2 are produced in higher amounts in most *C. acnes* strains. There are phylotype-specific variations; for example (some strains of) phylotype IB produce higher amounts of CAMP4 (Holland et al., 2010). CAMP2 and CAMP4 are abundantly secreted, as well as CAMP1; however, CAMP1 is also cell surface-exposed; it is predicted to be a sortase substrate (Girolamo et al., 2019). In human follicular casts CAMP1 was the most abundantly detected CAMP factor, followed by CAMP2 (Bek-Thomsen et al., 2014). The redundancy of CAMP factors is intriguing. *C. acnes* not only secretes high amount of CAMP factors but also decorates its surface with this protein. Thus, it can be assumed that CAMP factors have a vital function in the biology and/or host-interaction of *C. acnes*.

Regarding their functionality, CAMP factors are described as co-hemolysins, based on *in vitro* assays in which sheep blood erythrocytes are used (Christie et al., 1944). However, the *in vivo* function is likely to be quite different. Functional studies on CAMP factors of group B streptococci indicate that they are able to form micropores in eukaryotic membranes (Lang and Palmer, 2003). For the CAMP2 of *C. acnes* it was shown that is has cytotoxic effects in keratinocytes and macrophages if applied as recombinant protein (Nakatsuji et al., 2011). Another study reported that Toll-like receptor 2 (TLR-2) can recognize CAMP1 (Lheure et al., 2016). If confirmed, CAMP1 could be a trigger for an innate immune response when *C. acnes* comes in close contact with human (immunocompetent) cells. However, since *C. acnes* colonization of human skin usually does not go along with any detectable inflammation, the suspected adverse effects of CAMP factors, i.e., cytotoxicity and innate immune activation, must be tightly inhibited *in vivo*. The true *in vivo* function of CAMP factors might be different; e.g., it could be envisaged that CAMP factors, due to their pore-forming activity, could be involved in accessing nutrients within the sebaceous follicle microenvironment, without causing excessive damage to intact keratinocytes.

#### Lipases of *C. acnes* and Interaction With Host Glycolipids

Sebaceous glands produce sebum, a mixture of relatively non-polar lipids, in particular triglycerides, squalene, wax- and cholesterol-esters as well as free cholesterol. Sebum is usually overproduced during puberty due to the influence of androgens and other hormones on the sebaceous gland activity.

*C. acnes* has lipolytic properties; the bacterium possesses several lipases that can hydrolase triglycerides into fatty acids. A secreted lipase, the triacylglycerol lipase GehA, has been identified (Miskin et al., 1997). Interestingly, in human sebaceous follicles, another secreted lipase of *C. acnes* was detected in the infundibulum in larger amounts than GehA; this lipase was designated GehB (Bek-Thomsen et al., 2014). GehA and GehB are 42% identical on protein level.

The products of the activity of GehA/GehB, free fatty acids, have been suspected to contribute to acne. It is thought that alterations of the lipid composition in the infundibulum of the sebaceous follicle can provoke inflammatory responses (Zouboulis et al., 2014).

In addition to the hydrolysis of lipids, *C. acnes* possesses also several enzymes that can process glycolipids. Two putative endoglycoceramidases of *C. acnes* were identified within the infundibulum of sebaceous follicles (Bek-Thomsen et al., 2014). The proteins are predicted to catalyse the hydrolysis of the glycosidic linkage between oligosaccharides and ceramides of glycosphingolipids. As judged from the arsenal of enzymes that *C. acnes* possess, it can be hypothesized that the microorganism can process glycosphingolipids (e.g., gangliosides) to gain access to carbohydrates as nutrients: the oligosaccharide is removed from the ceramide by endoglycoceramidases; terminal sialic acid residues are removed by at least two sialidases (Brüggemann et al., 2004; Nakatsuji et al., 2008); the oligosaccharide moiety is further degraded by β-galactosidase, β-N-acetylhexosaminidase, endo-β-*N*-acetylglucosaminidase, and endo-α-*N*-acetylglucosaminidase. Only the latter enzyme, designated EngPA was characterized so far (Koutsioulis et al., 2008).

#### Short-Chain Fatty Acids (SCFAs)

Short-chain fatty acids are metabolites produced by *C. acnes* during fermentative growth; propionate, but also acetate, butyrate and valerate have been reported to be produced by *C. acnes*, in particular in the presence of glycerol in the growth medium (Shu et al., 2013b; Sanford et al., 2016; Tax et al., 2016). Diverse roles have been associated with the production of SCFAs by *C. acnes*. Shu et al. and Wang et al. reported that SCFAs can suppress the growth of *S. aureus* (Shu et al., 2013b; Wang et al., 2014a), indicating that *C. acnes* can prevent the colonization of *S. aureus* on the skin *via* the production of SCFAs. Nakamura et al. reported that SFCAs produced by *C. acnes* can inhibit biofilm formation by *S. epidermidis* by an unknown mechanism (Nakamura et al., 2020). Sanford et al. reported that SCFAs of *C. acnes* might have an adverse effect for skin barrier function: SCFAs (mainly produced under hypoxic conditions in the presence of lipids) inhibited histone deacetylase (HDAC) activity in keratinocytes; as a consequence, cytokine production in those keratinocytes was elevated in response to TLR2 ligands (Sanford et al., 2016). A similar observation was made in sebocytes (Sanford et al., 2019). In contrast, SCFA-mediated HDAC inhibition had the opposite consequence in monocytes (Sanford et al., 2016). Taken together, there seem to be health-beneficial as well as –detrimental roles of *C. acnes*-produced SFCAs. It is currently unknown, if this potentially fragile balance is influenced by the amount and/or the exact composition of SFCAs produced.

#### Porphyrins

*C. acnes* produce porphyrins as part of the vitamin B12 (cobalamin) cofactor biosynthesis pathway. They need cobalamin for essential enzymatic activities, e.g., for the activity of the methylmalonyl-CoA mutase, a key enzyme in the Wood-Werkman cycle that yields propionate production. Depending on the growth conditions and possibly depending on the individual strain, different precursor molecules can be accumulated during cobalamin synthesis, such as protoporphyrin IX, uroporphyrin III, and coproporphyrin III (Shu et al., 2013a). Under anaerobic conditions, protoprophyrin IX seems dominant, whereas under aerobic conditions, coproporphyrin III is more abundant. The amount of produced porphyrins might be strain-dependent: in general, type I strains produced more porphyrins compared to type II and type III strains (Johnson et al., 2016; Barnard et al., 2020). Porphyrins are suspected to have multiple effects: as an adverse effect, coproporphyrin III can induce *S. aureus* aggregation and possibly biofilm formation (Wollenberg et al., 2014). Others reported that coproporphyrin III can trigger cytokine responses in exposed cells (Schaller et al., 2005). However, clear mechanisms have not been unraveled so far. Among many open questions, it will be interesting to investigate the role of *C. acnes'* porphyrins on other members of the skin microbiome.

#### Biofilm

One of the main virulence traits associated with IAIs is biofilm formation of the respective pathogen in opportunistic infections (Tande and Patel, 2014). Several studies have proven the ability of *C. acnes* to develop a biofilm *in vitro*, using a range of materials (Bayston et al., 2007; Tafin et al., 2012) and in different types of implants such as hip or knee prosthesis, sternal wires (Holmberg et al., 2009) or cardiac pacemaker devices (Okuda et al., 2018). Also in AV, biofilm formation of *C. acnes* has been proposed as a mechanism that allows bacterial persistency and partial resistance to antimicrobial therapy (Coenye et al., 2007; Spittaels and Coenye, 2018; Aslan Kayiran et al., 2020). A *C. acnes* biofilm was also observed *in vivo* in sebaceous follicles with distinct *C. acnes* colonization patterns, such as the attachment of *C. acnes* to the follicular wall or matrix-encased biofilms localized in the infundibulum of the follicle (Jahns and Alexeyev, 2014). It should be noted here that *C. acnes* (biofilm) could not be detected in intact sebaceous glands (Alexeyev and Jahns, 2012; Jahns and Alexeyev, 2014). Scanning electron microscopy studies also revealed the formation of biofilm *ex vivo* on implants such as shunts (Bayston et al., 2007) or intraocular lenses (Suzuki et al., 2020b). A few studies about *C. acnes* biofilm formation in animal models were performed. Tafin et al. developed a foreign body-infection model in guinea pigs and results showed the ability of *C. acnes* to adhere to the implant surface. This study also demonstrated that rifampin was the most effective antimicrobial agent against sessile *C. acnes* (Tafin et al., 2012). As also proven for other biofilm-forming bacteria, sessile *C. acnes* cells seem more resistant to antimicrobials than planktonic cells; this could explain why *C. acnes*-associated IAIs often respond insufficiently to antibiotic therapy alone (Coenye et al., 2007; Tafin et al., 2012). Shiono et al. showed that *C. acnes* could survive in a biofilm for at least 6 months, causing delayed surgical site infection in a mouse osteomyelitis model (Shiono et al., 2016). An IAI rabbit model was developed by Achermann et al. (2015). The study also identified proteins of *C. acnes*, produced when grown in a biofilm and as planktonic cells. The protein signature of *C. acnes* in both states was similar; identified proteins were mainly from the core metabolism and some stress-related factors. This indicates that there is no large reprogramming taking place when *C. acnes* switches from a planktonic to a biofilm-embedded, sessile state. However, Coeyne et al. observed increased lipase production in sessile bacteria compared to planktonic cells (Coenye et al., 2007).

The biofilm-embedment may allow *C. acnes* to exist in a latent, dormant state that does not provoke a substantial inflammatory host response. However, specific mechanisms employed by *C. acnes* that would allow the formation and maturation of a biofilm have not been investigated in detail. Kuehnast et al. proposed that there might be a correlation between biofilm formation and the *C. acnes* phylotype, rather than the anatomical site of infection (Kuehnast et al., 2018). The study showed that strains belonging to phylotype IA_1_ formed higher amounts of biofilm in microtiter plates than other phylotypes. This is in accordance with Okuda et al.; they demonstrated that biochemical properties and structures of biofilms differed among *C. acnes* isolates (Okuda et al., 2018). In addition, it was suggested that extracellular DNA may play a role in the formation of *C. acnes* biofilm, as also seen for many biofilms produced by other bacteria. Gannesen et al. develop a method to analyze the composition of the biofilm matrix of *C. acnes*. They found that the major polysaccharide component of the matrix was the same as the cell wall polysaccharide (Gannesen et al., 2019). This indicates that *C. acnes* does not produce a specific extracellular polysaccharide, but rather aggregates *via* the existing cell wall polysaccharide.

Taken together, the current data suggest that *C. acnes* can form biofilms or aggregates. However, *C. acnes* seems not to be a dedicated, specialized biofilm-former, i.e., it does not use a specific program to form a differentiated biofilm, like it is seen in other species such as *ica*-positive staphylococci. Indeed, compared to other biofilm-forming bacteria, *C. acnes* biofilms are rather weak and easily to dissolve, at least *in vitro* (Kuehnast et al., 2018). Observed differences in biofilm formation in different studies may, at least in part, be related to variations in the employed biofilm detection method and materials used rather than represent real biological variation (Holmberg et al., 2009). It seems likely that *C. acnes* uses endogenous molecules for adhesive purposes, whose main functions are related to structural integrity, e.g., cell wall polysaccharide. Biofilm formation *in vivo* can be fortified by host-derived molecules such as fibrinogen, as *C. acnes* has fibrinogen-binding proteins (DsAs) on its surface (Grange et al., 2017; Petersson et al., 2018; Pickering et al., 2019).

#### Antimicrobial Resistance of *C. acnes*

Antimicrobial resistance (AR) of *C. acnes* is a major concern in acne but also in other *C. acnes*-associated diseases. A few reviews have summarized the current knowledge regarding the types of AR and the dissemination of resistant *C. acnes* strains (Eady et al., 2003; Dessinioti and Katsambas, 2017; Karadag et al., 2021). In brief, there is a large variation concerning the frequency of AR of *C. acnes* in different countries, which might be due to different treatment strategies, in particular regarding acne. In general, higher frequencies of AR are observed regarding erythromycin and clindamycin, while resistance to tetracyclines is usually lower. Long-term use of antibiotics is associated with increased macrolide resistance (Nakase et al., 2018). Resistance in *C. acnes* to relevant antibiotics is mainly due to point mutations in genes encoding ribosomal RNAs (16S rRNA and 23S rRNA genes; Lomholt and Kilian, 2014; Nakase et al., 2017). In addition, *erm*(X), encoding a RNA methylase that mediates resistance to clindamycin, has been identified on a mobile genetic element (Ross et al., 2002). Interestingly, a novel mobile genetic element of *C. acnes* was recently identified, conferring resistance to macrolides, clindamycin, and tetracyclines: the plasmid pTZC1 carries a novel macrolide-clindamycin resistance gene, *erm*(50), as well as a tetracycline resistance gene, *tet*(W) (Aoki et al., 2020). Resistant strains of *C. acnes* often belong to the acne-associated types, i.e., phylotypes IA_1_ (SLST types A and C) and IA_2_ (SLST type F) (Lomholt and Kilian, 2014; Nakase et al., 2017; Sheffer-Levi et al., 2020).

### Sensing and Signaling of Extra- and Intracellular *C. acnes* and Innate and Adaptive Immune Responses

*C. acnes* interacts on various levels with human cells such as skin-resident keratinocytes, but possibly also with sebocytes and with immune cells, including skin-resident Langerhans cells (LCs), dendritic cell (DCs), macrophages, and T cells (reviewed in Mayslich et al., 2021). Previous studies have shown, mostly in cell culture, that *C. acnes* has extensive immunostimulatory activity. The bacterium can stimulate the production of antimicrobial peptides and diverse chemokine and cytokines. For instance, in keratinocytes, activation of hBD2, TNF-α, GM-CSF, and IL-1α, IL-1β, and IL-8 has been observed upon bacterial encounter; in monocytes, *C. acnes* triggers the production of cytokines such as IL-1β, IL-8, IL-12, and TNF-α (Chen et al., 2002; Nagy et al., 2005; Yu et al., 2016). Innate host cell receptors can sense *C. acnes*, either extra- or intracellularly. Important for sensing extracellular bacteria is TLR-2, since TLR-2 activation is crucial for NF-κB activation in response to *C. acnes* (Kim et al., 2002; Su et al., 2017). As a TLR-2 agonist, CAMP factor 1 of *C. acnes* might play a role (Lheure et al., 2016). Intracellular recognition of *C. acnes* possibly involves other receptors, such as TLR-9 and NOD-like receptors, in a cell type-dependent manner (Kalis et al., 2005; Tanabe et al., 2006). In addition, *C. acnes* can activate the NLRP3 inflammasome, a system responsible for the activation of inflammatory processes *via* IL-1β maturation (Kistowska et al., 2014; Qin et al., 2014). A recent study has shown the involvement of another pathway in sensing intracellular *C. acnes*: bacterial DNA seems to be sensed in the host cell cytosol *via* the cGAS/STING pathway, resulting in a type I interferon response (Fischer et al., 2020). *C. acnes* likely interacts also with other immune cells such as T-cells. The bacterium can induce the production of T helper type 1(Th1)-type cytokines (e.g., IL-12, IFN-γ, and TNF-α) and Th17-type cytokines (e.g., IL-17 and IL-22) (Thielitz et al., 2008; Agak et al., 2014; Kistowska et al., 2015). In line with this, *C. acnes*-specific Th17 and Th1/Th17 cells can be detected in the peripheral blood of acne patients. Different *C. acnes* strains seem to have distinguishable abilities, for example to induce Th17-type cytokines; thus, *C. acnes* might modulate CD4^+^ T-cell responses in a strain-specific manner (Agak et al., 2018).

Taken together, the pro-inflammatory activity of *C. acnes* involves host cell signaling pathways and systems such as NF-κB, the inflammasome, and STING/cGAS. Less well-understood is which *C. acnes* components, e.g., genomic DNA, surface/secreted proteins, peptidoglycan and cell wall polysaccharide, are actually sensed and how the pro-inflammatory activity of *C. acnes* is controlled/dampened on normal skin. *C. acnes*-exposed keratinocytes may attenuate TLR-induced inflammation by negative regulatory circuits involving proteins such as TNIP1 and TNFAIP3 and microRNAs such as miR-146a, that can downregulate the production of inflammatory cytokines and chemokines (Erdei et al., 2018, 2021; Zeng et al., 2019). In addition, besides keeping bacteria in physical distance to immunocompetent cells, e.g., within infundibula of sebaceous follicles, another strategy to limited responses might be to eliminate invasive *C. acnes*. Intracellular bacteria are degraded by endocytosis/phagocytosis and in addition, the autophagic pathway is involved (Nakamura et al., 2016; Megyeri et al., 2018). It was suggested that induction of autophagy is caused by *C. acnes* cells in the host cell cytosol that escaped from endosomes (Nakamura et al., 2016). Another open question concerns the extent and significance of strain differences in host cell responses; here, knowledge remains fragmentary, since different researchers use often different strains. Thus, a robust, multi-laboratory investigation is needed with a defined panel of well-characterized *C. acnes* strains.

## Dr. Jekyll—Proven and Suggested Health-Beneficial Roles of *C. acnes*

### General Considerations

Since the first time Antonie van Leeuwenhoek set his eyes on bacteria in 1683, the scientific community has focused on understanding their role in pathogenesis, deciphering the host-pathogen interactions through molecular analysis of virulence factors and traits. This focus has paved the way to medical land-winnings including vaccines, antibiotics, and the general idea of hygiene. While a focus on bacterial pathogens and their molecular mediators of virulence has lent us much knowledge and medical tools, it has also to a large extent made us forget about the majority of bacteria, living in symbiosis with us, being commensal or mutualistic in their interaction.

**Figure 4:**
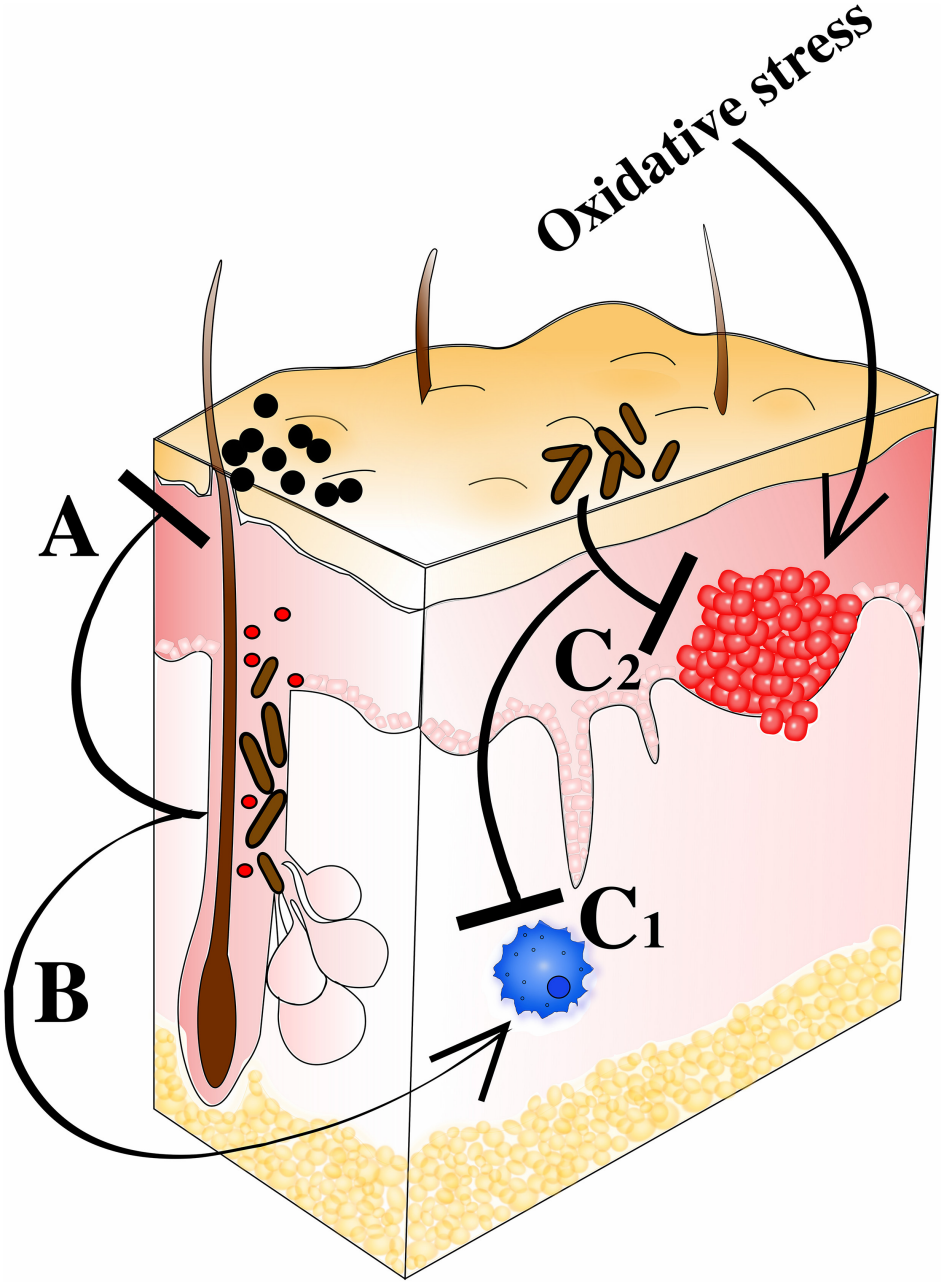
*C. acnes'* prospective probiotic qualities. (A) Colonization resistance against staphylococci and other potential pathogens. Direct effects through expression of SCFAs (e.g., propionic acid) and bacteriocins (e.g., cutimycin). Indirect effects through induction of host-derived AMPs (e.g., HBD and LL-37). (B) Modulation of immune response including e.g., Th1 and Th17 priming leading to heightened intrinsic anti-tumor activity and clearance of pathogens. Interactions with Langerhans cells also help shape host immune defenses. (C1) Inflammation regulation *via* RoxP, CLAs, polysaccharides, and SCFAs (pro- and anti-inflammatory qualities). (C2) Counteracting the tumorigenic effect of oxidative stress through RoxP secretion.

The idea of some bacteria being beneficial was raised several centuries ago, but has recently received increased attention (Fijan, 2014), and also turned more molecular (Mazmanian et al., 2008; Wang et al., 2014c; Wollein Waldetoft et al., 2020). Now it is well-recognized that (commensal) bacteria play a critical role in maintaining health and microbial dysbiosis being a cause to several common diseases (Harmsen and de Goffau, 2016; Zhu et al., 2018). We recognize the importance of gut bacteria for production of vital metabolic products (e.g., vitamin K; Conly and Stein, 1992) and how commensals produce bacteriocins and antibacterial substances in order to reduce the colonization of pathogens (Hammami et al., 2013), as well as how bacteria orchestrate the immune system to improve its defensive capacity (Naik et al., 2015). The raised awareness of beneficial aspects of the commensal microbiota has led to a re-evaluation of some of the bacterial members of the microbiota. In particular, the ubiquitous skin bacterium *C. acnes* has received much attention during the last years, being suggested to not only limit colonization by more potent pathogens (Wang et al., 2014a), but also to positively modulate the immune system (da Silva et al., 2013; Talib and Saleh, 2015), produce beneficial metabolites (Christensen and Brüggemann, 2014), treat and protect from tumor development (Tsuda et al., 2011), and maintain redox homeostasis on the skin (Allhorn et al., 2016). Here we summarize the documented beneficial effects of *C. acnes*, describe their molecular mechanisms, and discuss their impact in maintaining our health (Figure 4).

### *C. acnes* As a Regulator of Pathogenic Skin Colonizers

The commensal microbiota has been attributed many generic functions, including the ability to limit colonization by more pathogenic bacteria (Abt and Pamer, 2014). While undoubtedly so, the molecular mechanisms underlying such inhibition are mostly unknown. Production of antibacterial substances, reduction of nutritional availability and steric hindrance to adhere to the host have been suggested to be important factors (Silva et al., 2015). *C. acnes*, colonizing a highly specific niche of the skin, employs several mechanisms to promote its exclusivity in this environment.

#### Bacteriocins as Regulators of the Skin Microbiota

Competition for colonization not only exists between different bacterial species, but also between different bacterial strains. For that purpose, several bacteria produce bacteriocins that can target similar bacteria. Two different bacteriocins have been reported for *C. acnes*; acnecin and a thiopeptide, designated cutimycin. Acnecin was identified 1978, but no follow-up studies have been conducted since. The protein was determined to be a pentamer consisting of five 12 kDa monomers, exerting an antibacterial effect toward non-acnecin producing *C. acnes* strains, but not toward other species (Fujimura and Nakamura, 1978; Nakamura et al., 1978). Further characterizations concluded its protein composition, being negatively charged (pI 5.5) with a low carbohydrate content. Strikingly, acnecin was reported to be bacteriostatic only, not bactericidal (Fujimura and Nakamura, 1978).

The thiopeptide cutimycin has only recently been identified through comparative genomic studies (Brüggemann et al., 2012), uniquely found in type IB *C. acnes* strains only (Christensen et al., 2016). This peptide was shown to efficiently kill *S. epidermidis* and thus give *C. acnes* a competitive advantage to colonize the skin (Claesen et al., 2020). On a similar basis, *S. epidermidis* was also shown to be able to limit growth of *C. acnes* by several mechanisms and molecules; for example, fermentation products of *S. epidermidis* have been shown to mediate such reduction of fitness for *C. acnes* (Wang et al., 2014b). In contrast, Xia et al. demonstrated how *S. epidermidis* can locally reduce *C. acnes* induced inflammation, thus improving *C. acnes* fitness on the skin (Xia et al., 2016). This suggests that this microbe-microbe interaction is multifaceted.

#### Acidification by Propionic Acid Creates a Niche for *C. acnes*

A broader, less specific means to limit colonization by other bacterial species is mediated by the creation of highly specific niches. The former name of *C. acnes, Propionibacterium acnes*, relates to its ability to ferment sebum triglycerides and produce propionic acid. SCFAs, among them propionic acid, produced by *C. acnes* results in a lowered pH that is toxic to many microbes, both bacteria and yeast (Wang et al., 2014a). The ability of *C. acnes* to kill *S. aureus* through production of propionic acid has been proven both *in vitro* and *in vivo* (Shu et al., 2013b), and may partake in limiting *S. aureus* colonization on skin. Thereby, it was shown that the acidity *per se* is not toxic to the bacteria, but a high concentration of propionic acid itself (>25 mM) is efficiently killing several species. *C. acnes* itself is resistant to the inhibitory effect of propionic acid at biologically relevant levels (Wang et al., 2014a), allowing it to create an ecological niche on the skin, free of (most) other microbial species. The ability to produce propionic acid and to acidify the surrounding has however also been associated with the pathogenicity of *C. acnes* (Tax et al., 2016), making it a dual-edged sword.

#### Short Chain Fatty Acids Produced by *C. acnes* Promote a Healthy Microbiota

Besides the ability of *C. acnes* to produce propionic acid from sebum components such as triglycerides, a few other SCFAs (acetic acid, butyric acid, and valeric acid) can also be produced through fermentation (Shu et al., 2013b; Sanford et al., 2016). SCFAs have proven to limit growth and colonization of several skin pathogens (e.g., *S. aureus* and *Streptococcus pyogenes*) while not significantly affecting the growth of skin commensals (Gribbon et al., 1993; Hevia et al., 2015). Besides serving as a mean to limit pathogen colonization, the presence of SCFAs entail increased metabolic opportunities for the host (Morrison and Preston, 2016; LeBlanc et al., 2017). Furthermore, such SCFAs have been proven to positively regulate both inflammation and development of cancer (LeBlanc et al., 2017). It has been argued that the ability of *C. acnes* to produce such substances is a positive trait of a mutualistic lifestyle, stressing its benevolent nature (Christensen and Brüggemann, 2014).

### Beneficial Immunomodulating Properties of *C. acnes*

Since the discovery of *C. acnes* around 1,900 (Evans et al., 1950; Moore and Cato, 1963), its inflammatory and thereby immunomodulatory properties have been recognized. Initially seen as only a pathogenic trait of *C. acnes*, the induction of a Th1-type immune response has been taken advantage of for preventing, protecting, and treating several different pathologies.

#### *C. acnes* As an Adjuvant Reduces Sensitivity to Infections by Other Microbes

Due to the inflammatory properties of *C. acnes*, heat-killed *C. acnes* bacteria are commonly used as adjuvants in vaccines e.g., for horses, activating macrophages efficiently through its unique peptidoglycan structure (Peters and Hay, 1990; Paillot, 2013). However, injection of heat-killed *C. acnes* into a host has also been shown to affect sensitivity to infection by specific microbes as well as polymicrobial infections. While not being considered a zoonotic pathogen, the impact of *Actinobacillus pleuropneumoniae* on pig production is substantial (Li et al., 2013). Pre-treatment with *C. acnes* leads to a potent defense against *A. pleuropneumoniae* infections, likely due to cross-reactivity between one or several *C. acnes* antigens. A similar study in mice reached the same conclusion, showing a *C. acnes* dose-dependent protection toward *A. pleuropneumoniae* infection (Yang et al., 2014). Other studies have shown that pre-treatment of mice with heat-killed *C. acnes* allows for a higher survival rate (50 vs. 0%) when exposed to a polymicrobially induced sepsis (da Silva et al., 2013), indicating a more general priming of the immune system. Similarly, pre-treatment of mice with heat-killed *C. acnes* significantly improves infection with the parasite (worm) *Heligmosomoides polygyrus* (González-Sánchez et al., 2014). Thus, *C. acnes* may act as a general immunomodulator, orchestrating our immune defense against pathogens. In line with this, it was recently shown by Agak et al. how the presence of *C. acnes* can generate specific T_H_17 clones with a broad antibacterial activity, protecting against pathogens (Agak et al., 2021).

#### *C. acnes* Can Treat Solid Tumors

Due to the immunomodulatory effects of *C. acnes*, inducing a Th1 response, it was speculated if injections of *C. acnes* could shift Th2 responses toward Th1. A common Th2-driven disease is tumors, protecting themselves through modulating the immune response into a B-cell response (e.g., Th2) rather than a Th1-response that would be more apt to clear tumors through cytotoxic T-cells and NK cells (Disis, 2010). Historically, heat-killed *C. acnes* has been experimentally injected intra-tumorally in mice, resulting in significantly reduced pathology and tumor size (Bartlett et al., 1980; Kennedyl and Conley, 1989). Treatment of melanoma led to a switch in the immune response, both locally (skin) and systemically, to a Th1 response, resulting in heavy infiltration of T-cells to tumor lesions (Tsuda et al., 2011). In breast cancer a similar immunological change was observed, resulting in the complete removal of tumors in 40% of the mice, and significantly reduced angiogenesis while given in combination with melatonin (Talib and Saleh, 2015). With no reported toxicity and significant anti-tumor effect, the usage of *C. acnes* as an immunomodulator for tumor reduction may be a future therapy option.

#### Th1-Driven Response to *C. acnes* Restores Skin Pathologies

Several skin disorders have immunomodulatory pathologies, including atopic dermatitis and psoriasis (Griffiths et al., 2017). Both diseases have been reported to be associated with microbial dysbiosis, having significantly increased levels of *S. aureus* and reduced levels of *C. acnes* (Gao et al., 2008; Francuzik et al., 2018), and the presence of *C. acnes* has been suggested to play an important role in maintaining the skin health by influencing the skin microbiome (Chang et al., 2018). Supporting this claim, Kitagawa et al. demonstrated the protective effect of *C. acnes*, inducing a Th1 immune response locally and systemically, that resulted in significant improvement of atopic dermatitis in mice (Kitagawa et al., 2011). Similarly, the Th2-driven disease focal segmental glomerulosclerosis can be prevented, or limited if treated after onset, with the polysaccharide fraction of *C. acnes* (Reis et al., 2012). Whether the presence of *C. acnes* on the skin can protect from, or reduce the risk of, development of Th2-driven skin diseases is currently unknown. However, the current data suggest that this may be an interesting scientific avenue to pursue.

### Health-Beneficial Molecules Produced by *C. acnes*

Besides the abovementioned health-promoting aspects of *C. acnes*, several recent reports have indicated a direct health beneficial potential of *C. acnes*, by means of secreted molecules that may positively impact our health (Cogen et al., 2008). These benevolent factors may play a role in the protection from several diseases including oxidative skin diseases and skin cancer (He et al., 2015; Allhorn et al., 2016).

#### Conjugated Linoleic Acid (CLA)

Different variants of CLAs can have significant health-beneficial effects, including lowering cancer risks and enhanced immune defenses (Benjamin et al., 2015). Production of those acids are mainly through chemical reactions, resulting in a mixture of isomers, with some being more beneficial than others (Yang and Liu, 2004). However, it was recognized that *C. acnes* possesses a linoleic acid isomerase creating *trans*-10, *cis*-12 CLA as a single isomer (He et al., 2015) The biological relevance of the isomerase and the exact role of *trans*-10, *cis*-12 CLA on the skin have yet to be explored.

#### Antioxidant RoxP

Being a facultative anaerobe, *C. acnes* has developed several means to protect itself from oxidative stress, including catalase, peroxidases, and superoxide dismutase (Rolfe et al., 1978). Recently, it was discovered that *C. acnes* secrets high levels of the potent antioxidant RoxP. RoxP was found to be unique for *C. acnes*, conserved in all strains, and indispensable for an efficient colonization on skin (Allhorn et al., 2016). Due to an oxidative element in several skin pathologies the presence of *C. acnes* and RoxP could have protective functions and limit the negative effect of oxidative stress and development of disease.

Oxidation of skin, mediated by UVB irradiation, is a common cause of skin cancer (Narendhirakannan and Hannah, 2013). While *C. acnes* stimulate apoptosis of UVB-damaged melanocytes, other skin bacteria (e.g., *S. epidermidis*) promote their survivability (Talib and Saleh, 2015). Thus, not only may *C. acnes* prevent initial oxidative stress through secretion of antioxidants, but may also be capable of clearing cancerous skin cells.

Using RoxP as a biomarker for healthy skin, several highly sensitive biosensors have been developed (Ertürk and Lood, 2018; Ertürk et al., 2018; Ertürk Bergdahl et al., 2019), enabling absolute quantification of RoxP from skin samples from a diversity of skin conditions (healthy, actinic keratosis, basal cell cancer). This demonstrated the association of RoxP and *C. acnes* with oxidative diseases (Andersson et al., 2019). The ability of an ubiquitous skin bacterium to produce high quantities of an antioxidant, that aids in the protection from oxidative stress is of interest from both a biological and medical perspective. Modulation of the microbiota may thus be a means of affecting the redox homeostasis of the skin. Further studies are needed to properly evaluate the *in vivo* role of RoxP.

## Conclusions

Originally identified from acne, and historically being disreputed as a skin pathogen only, more and more evidence has enabled us to start understanding the complex roles of *C. acnes*. Recent research indicates that this species is a beneficial skin bacterium that fulfills important roles for skin homeostasis and protection. It also became clear that host responses to *C. acnes* can have both, beneficial and detrimental consequences. The criteria that decide about the outcome are multifactorial, and include, among others, the *C. acnes* phylotype/strain composition and population size, the tissue location of *C. acnes*, host tissue/cell tropism, the interaction with other skin bacteria, the predisposition and status of the host, including host genetics, as well as possibly other factors that are currently poorly understood, such as the influence of the gut microbiota.

Regarding the involvement of *C. acnes* in disease, it is widely accepted that *C. acnes* plays an important role in skin disorders such as AV, even though there are still gaps in our understanding of its exact contribution to disease. More and more studies point to a strong impact of the skin's immune system and the interactions of *C. acnes* with skin-resident immunocompetent cells. Thus, in order to understand the role of *C. acnes* in AV, one needs to understand the immune system of the skin.

Regarding the involvement of *C. acnes* in non-skin diseases there is a more controversial debate. One reason for the skepticism is connected to the omnipresence of *C. acnes*, which makes it difficult/impossible to apply Koch's postulates. It is difficult to exclude skin-derived contamination during sampling of the diseased tissue site; in this regard, DNA-based diagnostic tools applied on human specimens may be oversensitive, in particular regarding studies that use samples (e.g., tissue biopsies) with low microbial biomass (Eisenhofer et al., 2019). Thus, despite intensive research, our knowledge about the contribution of *C. acnes* to non-skin diseases is far from being complete. For IAIs, there has been an opinion change in the last decade; the scientific community now largely acknowledges that *C. acnes* can be a monomicrobial cause of IAIs, like other skin-resident bacteria such as coagulase-negative staphylococci. In Table 1 we have tried to evaluate the current evidence level for a (major) role of *C. acnes* in several diseases, based on clinical and experimental data.

Further understanding of *C. acnes'* biology is needed; in particular, we need to know more about its produced and secreted factors and their host-interacting functions in order to enable us to better recognize the beneficial and detrimental aspects of the bacterium and take advantage of them for improving (skin) health.

## Author Contributions

HB and RL wrote the main parts of the manuscript. LS-V and HG wrote specific sections. All authors contributed to the article and approved the submitted version.

## Conflict of Interest

HB, HG, and RL are members of the scientific advisory board of S-Biomedic. This had no influence on this work. The remaining author declares that the research was conducted in the absence of any commercial or financial relationships that could be construed as a potential conflict of interest.
